# Bone marrow derived mesenchymal stem cells ameliorate inflammatory response in an in vitro model of familial hemophagocytic lymphohistiocytosis 2

**DOI:** 10.1186/s13287-018-0941-y

**Published:** 2018-07-18

**Authors:** Handan Sevim, Yusuf Çetin Kocaefe, Mehmet Ali Onur, Duygu Uçkan-Çetinkaya, Özer Aylin Gürpınar

**Affiliations:** 1 Department of Biology, Faculty of Science, Hacettepe University, 06800 Ankara, Turkey; 2 Department of Medical Biology, Faculty of Medicine, Hacettepe University , 06100 Ankara, Turkey; 3 Department of Stem Cell Sciences, Institute of Health Sciences, Center for Stem Cell Research and Development (PEDI-STEM), Hacettepe University, 06100 Ankara, Turkey; 4 Pediatric Hematology, BMT Unit, Children’s Hospital, Hacettepe University, 06100 Ankara, Turkey

**Keywords:** Familial hemophagocytic lymphohistiocytosis 2, Perforin, Mesenchymal stem cells, CRISPR/Cas, Immune modulation

## Abstract

**Background:**

Familial hemophagocytic lymphohistiocytosis 2 (FHL2) is the most common familial type of hemophagocytic lymphohistiocytosis with immune dysregulation. FHL2 patients have mutations in the perforin gene which cause overactivation and proliferation of cytotoxic T lymphocytes and natural killer cells. Perforin is the key component of the cytolytic granule response function of cytotoxic T lymphocytes and natural killer cells. Perforin dysfunction causes a cytotoxic immune deficiency with a clinical outcome of uncontrolled and continuous immune stimulation response. This excessive stimulation leads to continuous systemic inflammation and, ultimately, multiorgan failure. Radical therapy is hematopoietic stem cell transplantation which is limited by the availability of a donor. Exacerbations of inflammatory attacks require a palliative immunosuppressive regimen. There is a need for an alternative or adjuvant therapy to maintain these patients when immunosuppression is ineffective or a donor is not available. Beneficial actions of mesenchymal stem cells (MSCs) have been shown in autoimmune diseases in clinical trials and are attributed to their immune-modulatory properties. This study aimed to assess the immune-modulatory effect of MSCs in an in-vitro model of FHL2.

**Methods:**

We generated a targeted mutation in the perforin gene of NK92 cells to create an in-vitro FLH2 model using Crispr/Cas technology. A coculture setup was employed to assess the immunomodulatory efficacy of MSCs.

**Results:**

Engineered NK92 clones did not show *PRF1* mRNA expression and failed to secrete perforin upon phorbol myristate acetate–ionomycin stimulation, providing evidence for a valid FHL2 model. Coculture media of the engineered cells were investigated for the abundance of several cytokines. Coculture with MSCs revealed a reduction in major proinflammatory cytokines and an induction in anti-inflammatory and immunomodulatory cytokines compared to the parental NK92 cells.

**Conclusions:**

This study shows the ameliorating effect of MSCs as an adjuvant immune modulator toward the therapy of FHL2 patients. MSCs are supportive therapy candidates for FHL2 patients under circumstances where prolonged immunosuppression is required to gain time before allogeneic hematopoietic stem cell transplantation.

**Electronic supplementary material:**

The online version of this article (10.1186/s13287-018-0941-y) contains supplementary material, which is available to authorized users.

## Background

Hemophagocytic lymphohistiocytosis (HLH) is a rare, severe, genetic immunodeficiency disease with an estimated incidence of 1.2 cases in 1 million. However, due to the high consanguinity rate in Turkey, the incidence of this rare disorder is much higher at about 7.5 of 10,000 hospitalized pediatric patients [1, 2]. Familial hemophagocytic lymphohistiocytosis 2 (FHL2) is the most common familial type of HLH with immune dysregulation. FHL2 patients have mutations in the perforin gene which cause overactivation and proliferation of cytotoxic T lymphocytes and natural killer (NK) cells [3]. Perforin is the key component of the cytolytic granule response function of cytotoxic T lymphocytes and NK cells [4]. As a pore-forming protein, perforin creates pores at the target cell membrane, inducing apoptotic death via granzymes [5].* PRF1* gene mutations cause perforin protein dysfunction, resulting in cytotoxic immune deficiency. The loss of cytotoxic immune function causes uncontrolled and continuous immune stimulation response accompanied with high levels of cytokine release in FHL2 patients [6, 7]. Uncontrolled stimulation of the immune system and excessive cytotoxic T-cell and NK-cell stimulation cause systemic inflammation and multiorgan failure [8].

The primary focus of HLH therapy is to suppress the overactivated immune system. The first line of palliative immunosuppressive therapy for HLH is defined by the international HLH2004 protocol and recommends administration of dexamethasone, cyclosporine, and etoposide in an 8-week course. Radical therapy for HLH is hematopoietic stem cell transplantation for a complete recovery, which is limited by the availability of a suitable HLA-compatible donor [9, 10]. However, unavailability of a suitable donor at the end of the 8 weeks of immunosuppressive therapy leaves the patient and the physicians without a choice until a second exacerbation. There is a need for an alternative or adjuvant therapy to maintain these patients when the immunosuppressive course is ineffective or a donor is not available.

Mesenchymal stem cells (MSCs) harbor immune-modulatory properties that are attributable to low expression of MHC class II antigens as well as cytokine secretion [11, 12]. Clinical trials and in vivo studies have shown beneficial immune-modulatory action of MSCs on autoimmune diseases [13–16]. In one unique report, Mougiakakos et al. [17] reported the administration of MSCs as an immune-modulatory approach for a single FHL3 patient with a beneficial outcome. However, a cell-based in vitro model is required for the assessment of this approach and to provide proof-of-concept results toward the beneficial impact of MSCs on FHL2. In this context, since primary cells from untreated patients are not available, this study was designed to assess the immune-modulatory effect of MSCs on the FHL2 in vitro model.

## Methods

### Isolation and characterization of human bone marrow mesenchymal stem cells

Human bone marrow MSCs were isolated from adult bone marrow aspirates from healthy bone marrow transplantation donors following written informed consent (Hacettepe University Local Ethical Committee approval #LUT12/134–16). Mononuclear cells were isolated by Ficoll density gradient and cultured with Dulbecco’s Modified Eagle’s Medium-Low Glucose (DMEM-LG; Biochrom, Germany)/MCDB201 (Sigma, USA) supplemented with 10% fetal bovine serum (FBS; Biochrom, Germany), 1% penicillin–streptomycin (Biochrom, Germany), and 2 mM l-glutamine (Biochrom, Germany) in humidified air with 5% CO_2_ at 37 °C. Characterization of the MSCs was done as reported previously in accordance with the guidelines of the International Society for Cellular Therapy [18, 19]. MSCs were characterized by flow cytometry (FACS Aria; Becton Dickinson) using an antibody panel that consisted of CD29, CD44, CD73, CD90, and CD105 as mesenchymal markers. In order to exclude hematopoetic lineages, CD3, CD34, and CD45 were investigated as negative markers. For further characterization, MSCs were differentiated toward osteogenic and adipogenic lineages as described previously [19]. Briefly, induction of osteogenic differentiation was achieved by supplementation of the media with dexamethasone, ascorbate, and β-glycerol phosphate for 21 days and differentiation was confirmed by Alizarin Red staining. Adipogenic differentiation was induced by incubation for 3 days in adipogenic induction medium that consisted of 1-methyl-3-isobutylxanthine, dexamethasone, insulin, and indomethacin. Lipid-rich vacuoles were evident in cells after 7 days, and were confirmed with Oil red O staining. Cells were observed using an Olympus IX70 inverted microscope equipped with an Olympus DP71 digital camera (Olympus, Japan).

### Perforin gene targeting using the CRISPR/Cas gene editing approach

The human NK cell line (NK92) was purchased from ATCC (CRL2407; ATCC, USA) and cultured in alpha-minimum essential medium (α-MEM) (Biochrom, Germany) supplemented with 100 U/ml IL-2 (Merck Millipore, Germany), 0.2 mM myo-inositol (Applichem, Denmark), 0.02 mM folate (F8758; Sigma, USA), 0.1 mM 2-β-mercaptoethanol (Sigma, USA), 12.5% FBS (Biochrom, Germany), and 12.5% Horse Serum (HS-Biochrom, Germany) in humidified air with 5% CO_2_ at 37 °C. Cells were passaged every 3 days at a ratio of 1:2.

CrispR/Cas9 genome editing technology was employed to target the *PRF1* gene (ENSG00000180644). Three different guide RNAs were cloned into the CRISPR/Cas plasmids pX335-U6-Chimeric_BB-CBh-hSpCas9n (D10A) and pX330-U6-Chimeric_BB-CBh-hSpCas9 (Addgene plasmids #42335 and #42230) that target exon 2 (ENSE00001614299) of the *PRF1* gene as described previously [20]. Furthermore, a donor plasmid was designed and prepared that flanked the exon 2 genomic sequence and contained human ubiquitin C promoter (PMID: 8650001) driving the expression of a puromycin resistance gene. Donor plasmid flanking arms encompassed at least 600 bases of the *PRF1* exon 2 sequence. Plasmid sequences were verified by Sanger sequencing.

Both plasmids were transfected into NK92 cells using an Amaxa 4D-Nucleofector and Cell Line Optimization 4D-Nucleofector™ X Kit (Lonza, Switzerland). Beginning 24 h following gene transfer, transfected cells were selected with 3 μg/ml puromycin-supplemented media for 4 weeks. Selected clones were verified for the targeting of the exon 2 by PCR screening using primers flanking exon 2 of the* PRF1* gene (forward, 5′-CAGACCCCTCCCTAAACCTG-3′; reverse, 5′-ATGATTGAAGCTCAGAGAGAA-3′).

### Functional validation

Selected clones were assessed at the functional level by stimulating with both PMA and ionomycin (50 ng/ml and 1 μg/ml, respectively) for 4 h. Perforin gene expression was investigated using quantitative PCR (qPCR) analysis and perforin protein levels were measured with ELISA assay. Total RNA (1 μg) from each selected clone was reverse transcribed into cDNA, and qPCR analysis was performed as described previously [21] using oligonucleotide primers targeting *PRF1* cDNA (forward, 5′-ACTTTGCAGCCCAGAAGACCCA-3′; reverse, 5′-CCAGCTCCACAGCCCGGAT-3′). qPCR amplification was normalized to the expression of the TATA binding protein (forward, 5′-TGCTGAGAAGAGTGTGCTGGAG-3′; reverse, 5′-ATTGGTGTTCTGAATAGGCTGTGG-3′). Perforin protein secretion from the selected clones was assessed in the cell supernatants using a perforin ELISA kit (Catalog No. ab46068; Abcam, UK) in accordance with the manufacturer’s protocol. Elisa plates were quantified at 450 nm as a primary wavelength with a microplate reader (EZ Read 400 Microplate reader; Biochrom, UK). All samples were run in triplicate, and the minimum detectable perforin level was 40 pg/ml.

### Coculture and multiplex cytokine assay

In order to assess the immunomodulatory effect of MSCs, selected clones and NK92 cells were stimulated with PMA and ionomycin (50 ng/ml and 1 μg/ml) for 4 h. Then, stimulated and unstimulated cells were transferred using a ThinCert™ six-well-plate transwell system (0.4 μm pore size; Greiner, Germany) onto six-well plates that were seeded with MSCs (1 × 10^4^ cells per well) 24 h a priori. This approach yielded a coculture setup at a ratio of 1:1 and was maintained up to 72 h.

Following incubation for 24 and 72 h, multiplex cytokine assay was performed on coculture supernatants. The Bio-Plex Pro Human Cytokine 17-Plex Immunoassay (Catalog No. M5000031YV; Bio-Rad, USA) was used to assess IL-1β, IL-2, IL-4, IL-5, IL-6, IL-7, IL-8, IL-10, IL-12, IL-13, IL-17, G-CSF, GM-CSF, IFN-γ, MCP-1 (MCAF), MIP-1β, and TNF-α levels. All procedures were carried out according to the manufacturer’s protocol and a Bio-Plex MAGPIX multiplex reader (Bio-Rad, USA) was employed. All experiments were performed in triplicate.

### Statistics

All values were expressed as mean ± standard deviation of biological replicates. Student’s *t* test was used to assess the significance of differences when two groups were compared. When more than three groups were analyzed, an ANOVA test was used to determine the significance between the groups followed by Tukey’s HSD post-hoc analysis (SPSS version 23.0 for Windows; SPSS, Chicago, IL. USA). A *p*-value cutoff of less than 0.05 was considered significant. The distribution of variation of the multiplex cytokine assessment results on all groups was visualized using the principle component analysis (BRB Array tools, V4.6.0 Beta_1 July 2017).

## Results

### Characterization of MSCs

Flow cytometric analysis of the cell surface antigen expression of MSCs revealed positive expression for CD29, CD44, CD73, CD90, and CD105 compatible with mesenchymal origin; and negative expression for CD34, CD45, and CD3 that excluded a hematopoietic lineage. Furthermore, the osteogenic and adipogenic lineage differentiation capacities of MSCs were also validated. Cytometry analysis showed that the MSCs fulfilled the determinant criteria of the International Society for Cellular Therapy [18]. Immunophenotyping and the results of differentiation experiments are presented in Additional file 1: Figures S1 and S2.

### *PRF1* gene targeting in NK92 cells and functional validation

**Fig. 1:**
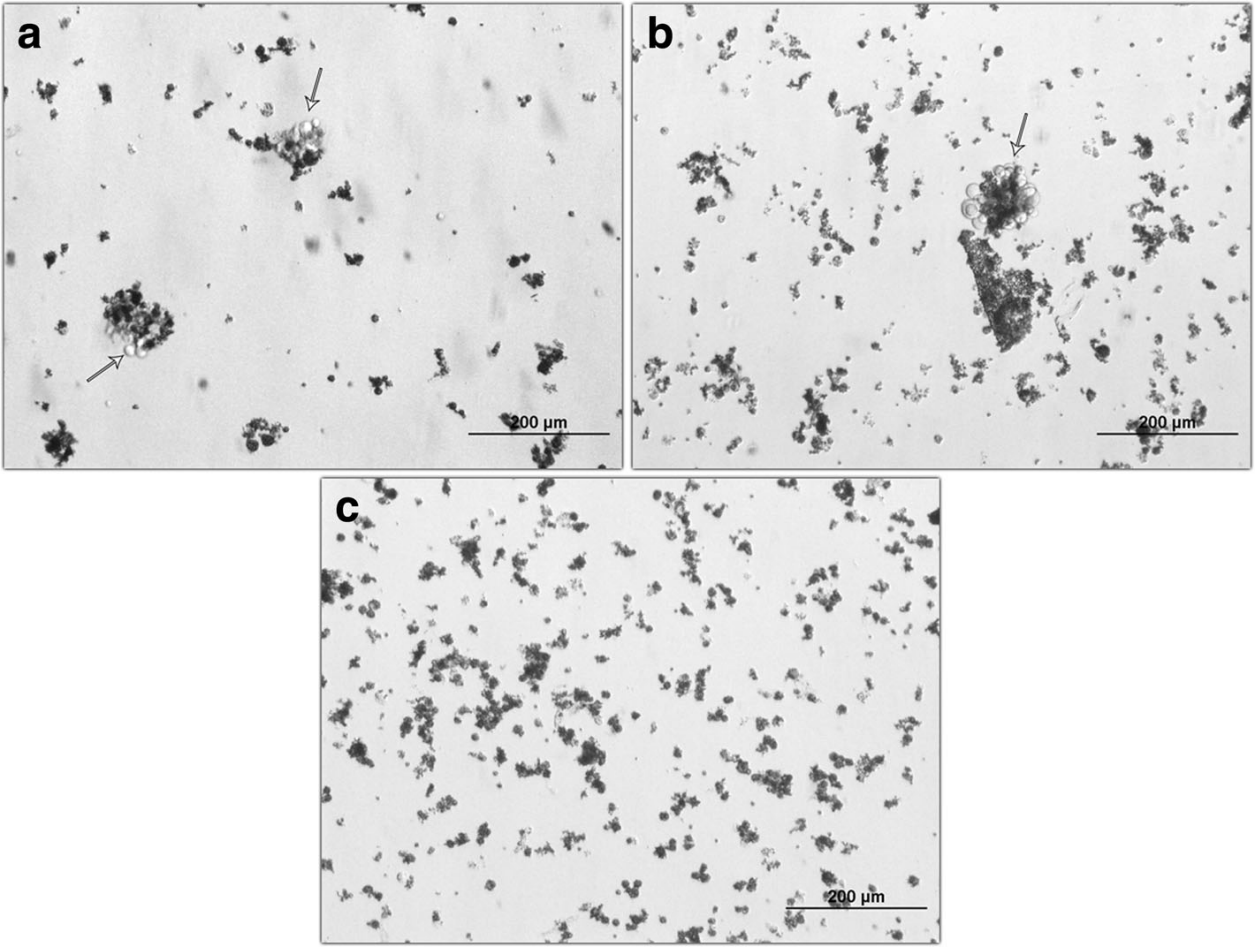
NK92 cells were cotransfected with CRISPR/Cas and donor plasmid vectors targeting *PRF1* gene. Cells selected in puromycin-containing media (3 mg/ml). Selected clones were screened for *PRF1* locus and two positive clones designated clone 1 (**a**) and clone 2 (**b**) were employed in the study. Arrows show selected and proliferating colonies. No surviving cells visible in nontransfected control group (**c**). Phase-contrast microscopy, scale bar = 200 μm

**Fig. 2:**
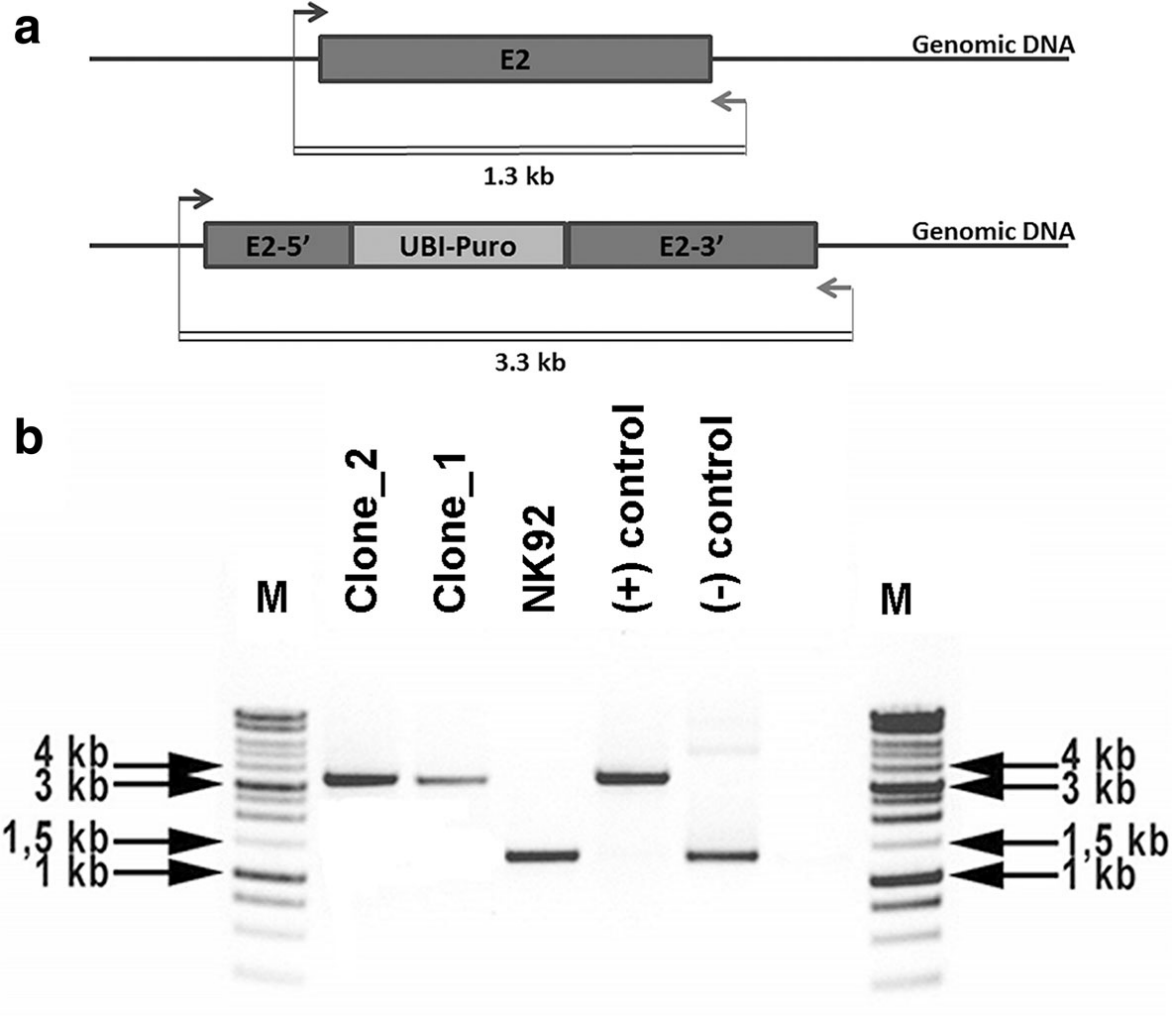
PCR screening for validation of selected clones. PCR primers flanking *PRF1* gene exon 2 designed to yield a 1.3-kb amplicon in nontargeted (wild-type) genome. Homozygous insertion of an expression cassette containing human ubiquitin C promoter driving the puromycin resistance gene yields a 2-kb amplicon (**a**). Analysis of PCR results on 1% agarose gel confirmed homozygous 2-kb insertion into *PRF1* exon 2 in selected NK92 clones (**b**). Donor plasmid vector used as positive control and human control DNA as negative control

**Fig. 3:**
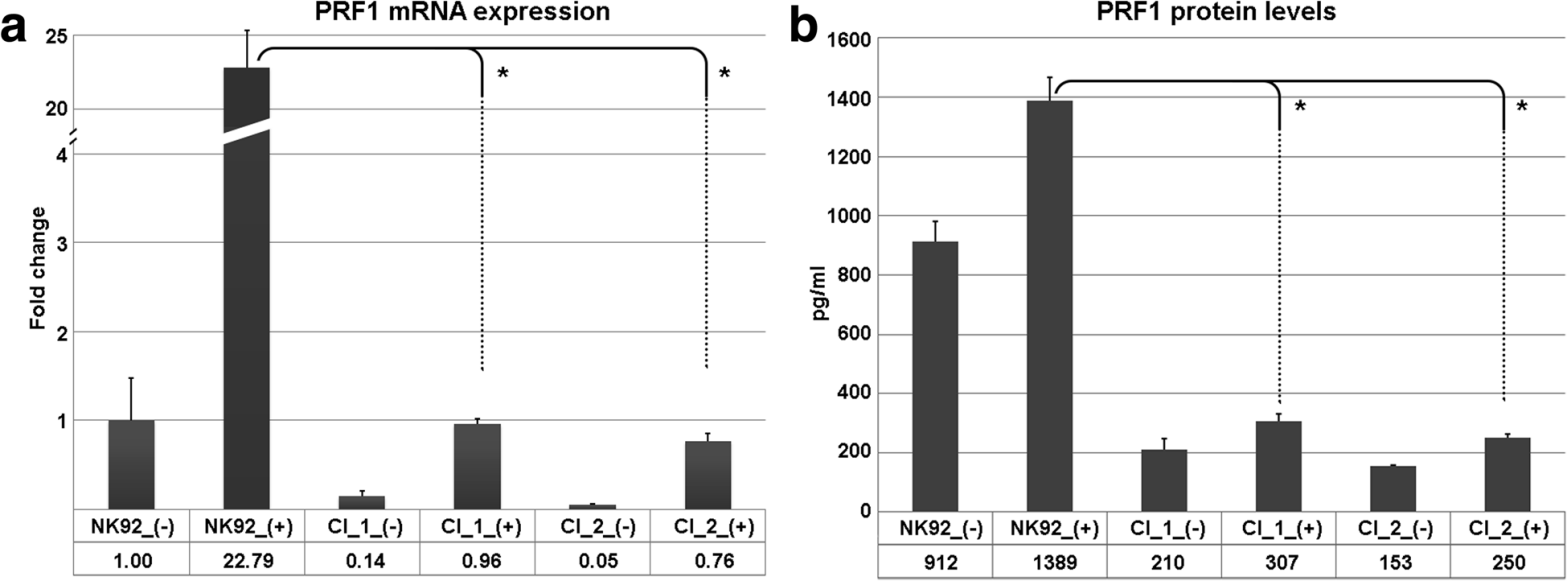
Functional validation of selected engineered NK92 clones verified by qPCR and ELISA. Targeted clones did not show an induction of *PRF1* mRNA expression following stimulation with PMA and ionomycin (**a**). Likewise, perforin protein analysis by ELISA indicated a similar profile. Upon stimulation, control NK92 cells (parental cells) secreted several fold of perforin. However, selected clones did not exhibit any significant change (**b**). Mean values of biological replicates shown, error bars show standard deviation. (+) indicates stimulated, (-) indicates unstimulated cells. *p < 0.05, Student’s *t* test; *n* = 6

Transfected cells were selected in cell culture media supplemented with 3 mg/ml of puromycin for 4 weeks. Several clones were screened for the targeting of *PRF1* locus and two positive clones were designated clone 1 and clone 2 (Fig. 1). A PCR screening strategy was employed for the targeting of exon 2 of the *PRF1* gene (Fig. 2a). The two selected clones exhibited homozygous insertion of the puromycin resistance gene construct (2 kb), revealing a total PCR product of 3.3 kb (Fig. 2b). Nontargeted NK92 cells revealed the wild-type *PRF1* exon 2 size of 1.3 kb. Functional validation of the clones was assessed via stimulation with PMA–ionomycin. Both *PRF1* mRNA and perforin protein levels were checked by qPCR and ELISA, respectively. qPCR results showed that stimulated control cells exhibited a remarkable *PRF1* upregulation of up to 20-fold compared to stimulated clone 1 and clone 2 cells (Fig. 3a). The secreted perforin levels were measured following stimulation. Control cells revealed 1389 pg/ml perforin while stimulated clones 1 and 2 were measured as 307 pg/ml and 210 pg/ml, respectively. Statistical analysis showed that the difference was extremely significant, *p* < 0.001 (Fig. 3b).

### Immune-modulatory effect of MSCs on FHL2 model

**Fig. 4:**
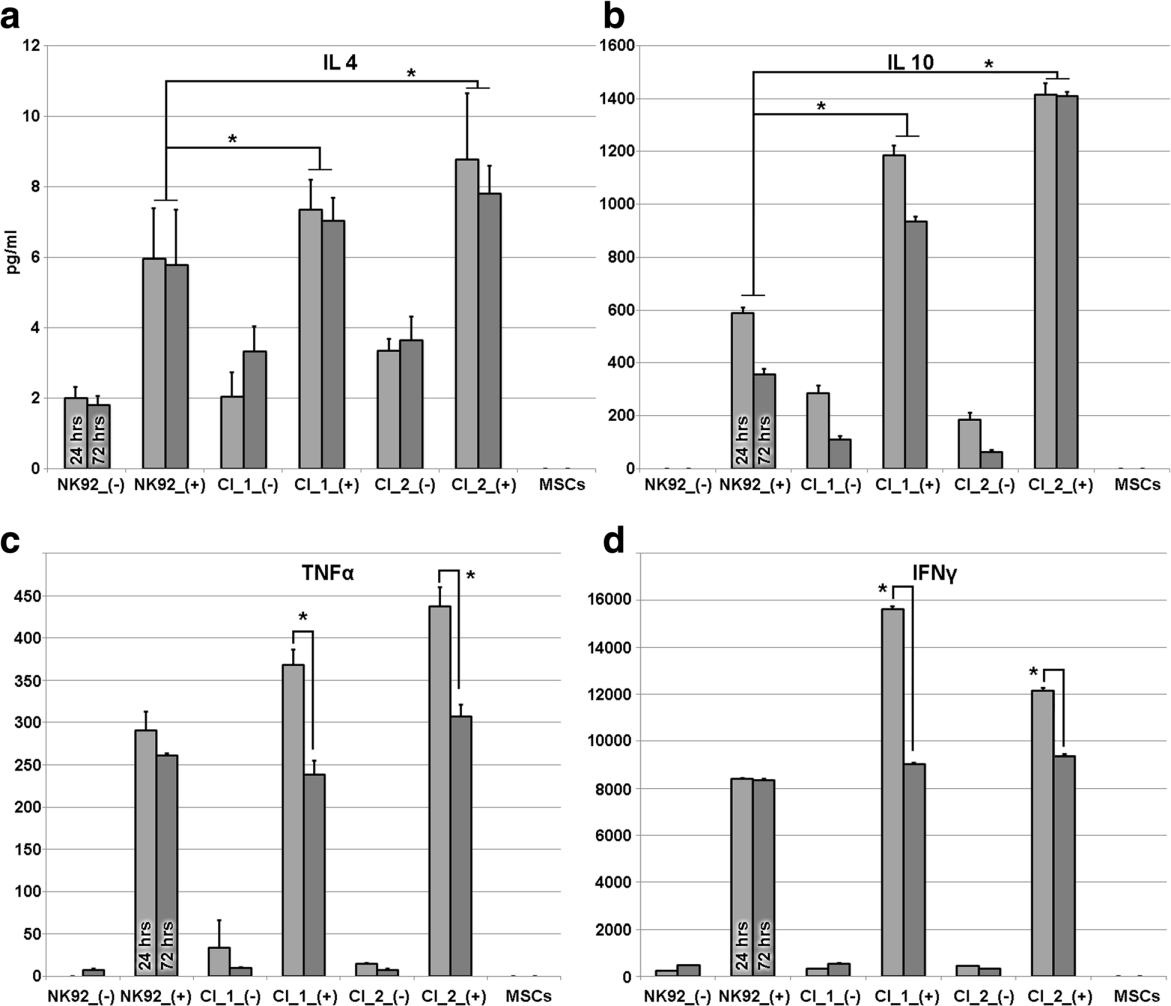
Multiplex assay employed to measure levels of several cytokines in coculture supernatants. When cocultured with engineered NK92 clones, MSC-derived immunosuppressive cytokine (IL-4 and IL-10) levels were higher, compared to parental NK92 cells (**a**, **b**). Major proinflammatory cytokines TNF-α and IFN-γ were significantly diminished at 72 h versus 24 h in supernatants of cocultured cells but not in stimulated parental NK92 cells (**c**, **d**), exhibiting a time-course suppression of activation when cocultured with MSCs. (+) indicates stimulated, (-) indicates unstimulated cells. Indicated cytokine levels in pg/ml. Mean values of biological replicates shown, error bars show standard deviation of *n* = 3. ANOVA used to analyze statistical significance among groups. *p < 0.05. IL interleukin, IFN interferon, MSC mesenchymal stem cell, TNF tumor necrosis factor

A coculturing approach was pursued to investigate the immune-modulatory effect of MSCs on stimulated and unstimulated clones. A multiplex cytokine assay was employed to measure IL-2, IL-4, IL-6, IL-8, IL-10, G-CSF, GM-CSF, IFN-γ, MIP-1β, and TNF-α cytokine levels. The cytokines that were absent in the culture supernatant (not secreted by the NK92 cells) were excluded from the analysis (IL-1β, IL-5, IL-7, IL-12, IL-13, IL-17, MCP-1). Results showed that levels of the major immune-modulatory cytokines IL-4 and IL-10 were significantly increased compared to stimulated parental NK92 cells (Fig. 4a, b). The major proinflammatory cytokines IFN-γ and TNF-α were observed to be significantly suppressed over the incubation period in clones cocultured with MSCs (Fig. 4c, d).

G-CSF and IL-8 cytokines are solely secreted from MSCs. While G-CSF levels show up to 4-fold increase upon stimulation in all cocultures, no significant variance was observed between the parental cells and clones; IL-8 levels were steady in all experiments regardless of stimulation or time. The source of GM-CSF, MIP-1β, and IL-6 in culture supernatant is both the NK92 cells and MSCs. In all stimulated cocultures, GM-CSF levels exhibited a similar increase over time. However, MIP-1β and IL-6 levels did not exhibit any variance in any of the observation groups (data not shown). IL-2 levels were not analyzed since NK92 medium was supplemented with IL-2.

## Discussion

FHL2 is a fatal immunodeficiency condition, caused by *PRF1* gene mutations exhibiting manifestations in immune cells that lead to systemic inflammation and multiorgan failure [22]. The FHL2 gene product perforin is stored in the cytoplasmic lytic granules of the immune cells and contributes to cytotoxic immune response [23]. Thus, absence of perforin leads to immune deficiency complicated by uncontrolled and prolonged release of cytokines by NK and cytotoxic T cells. Episodic attacks of life-threatening cytokine storms are the major clinical manifestation of FHL2. Allogeneic bone marrow transplantation is the only radical cure for FHL2 and a palliative immunosuppressive regimen is required a priori. However, not all cases respond to this immunosuppressive regimen. MSCs can be considered as an alternative adjuvant to the immunosuppressive protocol. This study was performed to determine the beneficiary immunomodulatory effects of MSCs in FHL2. An in-vitro model is required to validate this axiom. However, primary cells from an early-diagnosed and untreated patient are inaccessible for this rare disease [24]. Thus, CrispR/Cas9 genome editing technology was employed to generate a perforin knockout human NK cell line. Among several leukemic cell lines, NK92 can be considered a valid model since it has been shown to exhibit cytotoxic response to tumor cell lines, pathogens, and virus-infected cells through the perforin–granzyme pathway [25, 26]. The NK92 cytokine repertoire contains growth factors, proinflammatory cytokines, and chemokines [27]. The aforementioned properties directed us to choose NK92 as a suitable cell model.

**Fig. 5:**
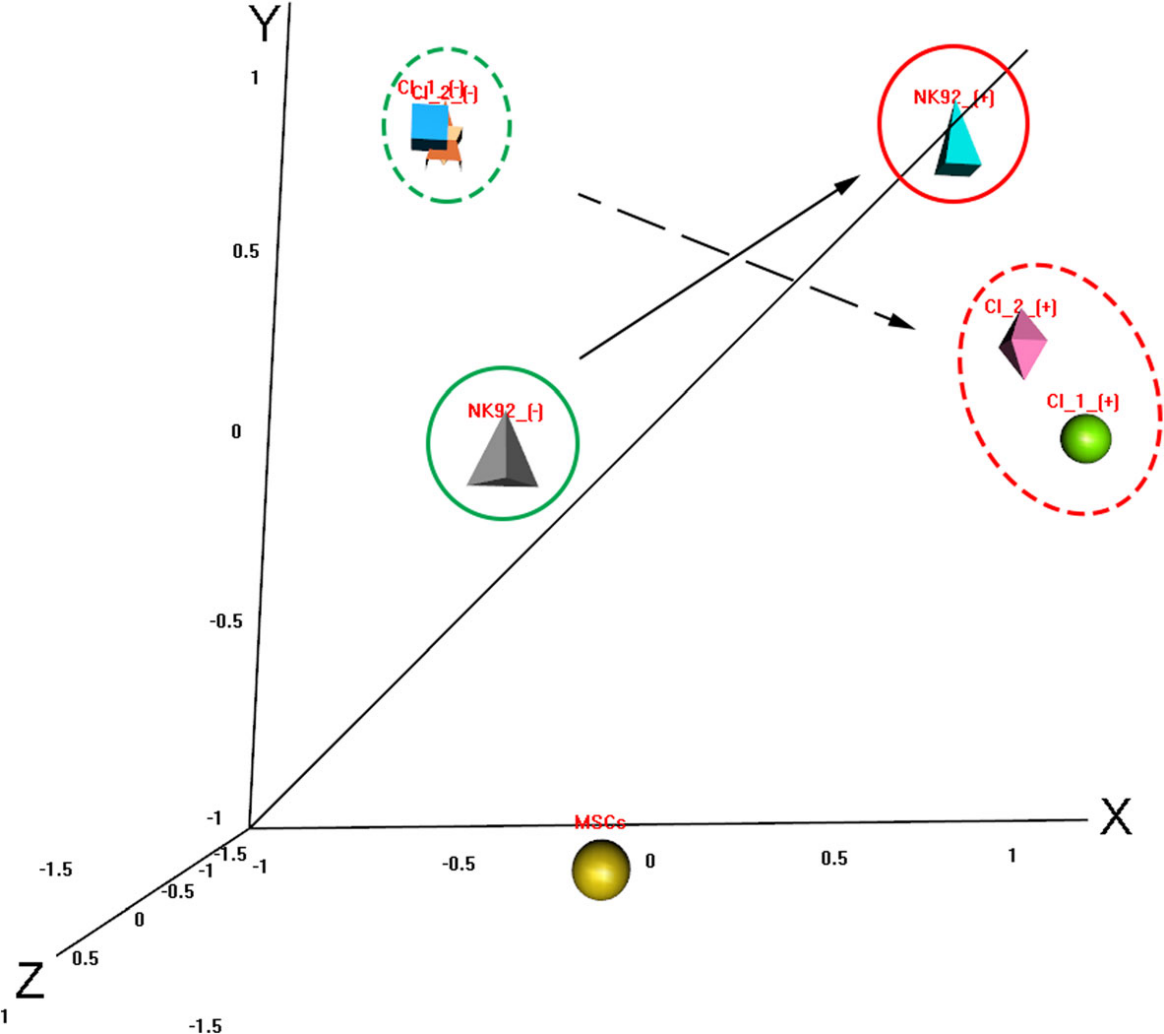
Principal component analysis employed to visualize and emphasize cytokine measurement data from MSC coculture experiments. Multidimensional representation of data depicts distinctive vectoral positioning of MSC coculture effect on parental NK92 cells (straight lines) versus engineered NK92 clones (dashed lines) following stimulation (red). MSC mesenchymal stem cell

The genetically engineered NK92 clones did not show *PRF1* mRNA expression and failed to secrete perforin upon PMA–ionomycin stimulation, providing evidence for a valid FHL2 model. The NK cells’ behavior repertoire upon stimulation is well known and the cytokine profile observed in this study is highly similar to previously published reports [28–32]. Since the primary aim of this study was to assess the effect of the presence of MSCs, the conducted cytokine experiments were designed to observe variances between NK92 cells and the engineered clones. Coculture of the engineered NK92 clones with MSCs exhibited significantly diminished expression of the major proinflammatory cytokines IFN-γ and TNF-α, and positively altered the expression of immunomodulatory cytokines IL-4 and IL-10. It was previously reported that MSCs secrete IL-4, IL-10, HGF, IDO, PGE_2_, and sHLAG5 [33, 34]. Here we observed an induction of IL-4 and IL-10, which are the major contributors of the attributed immunomodulatory action of MSCs [35]. IL-4 is solely secreted by MSCs only upon coculture with NK92 cells and IL-10 is secreted by both NK92 cells and MSCs, but a higher level of the latter is observed in cocultures. IL-10 was shown to potentiate a reduction in disease progression and severity in the MSC-administered mouse model of GVHD [36]. These results provide mechanistic evidence for the basis of the suppression of proinflammatory cytokine release. This action is best reflected in the IFN-γ levels followed by TNF-α. Both of these cytokines exhibit a time-course decrease over the observation period following stimulation. Furthermore, coculture with MSCs exhibited higher GM-CSF and G-CSF levels in the medium regardless of the coculture period. GM-CSF is known to inhibit the differentiation of monocytes into dendritic cells in the presence of IL-4 [37] and G-CSF was shown to reduce the cytotoxic activity of NK92 cells [38]. Therefore, the upregulation of these two cytokines in cocultures may also contribute to the observed immunomodulatory action compared to the parental NK92 cells. Principal component analysis (PCA) is a statistical technique used to visualize and emphasize multidimensional variation in a dataset. PCA is often used to make data easy to explore and visualize. We pursued this approach to reflect the impact of multidimensional cytokine measurement data from the MSC coculture experiments in a graphical context. The results showed a clear contrast between the MSC cocultured clones and the NK92 cells based on the complexity of the data (Fig. 5). Regarding the interaction of MSCs with NK cells, other questions need to be investigated beyond the effect of soluble factors. It is known that cell-to-cell contact and crosstalk involving nanotubular transport may also influence the immunomodulatory action of MSCs on NK and T cells [39, 40]. Thus, the experimental design using the transwell approach is solely suitable to observe the impact of soluble factors. Likewise, for FLH2 in a proinflammatory environment, the immunomodulatory function of MSCs on NK cells was previously attributed to soluble factors particularly in the presence of IFN-γ [41]. The functional assays presented in this study are complementary to the current knowledge base that is required before considering any clinical applications involving MSCs [42–44].

FHL2 is a rare, hard-to-diagnose disease, especially when present in the newborn. It is not uncommon to diagnose the disease after the loss of the patient [24]. With a timely diagnosis, an immunosuppressive treatment regimen is initiated immediately, particularly when the patient is in the active phase. Thus, primary NK cells from untreated and newly diagnosed patients are very difficult to obtain. Engineered NK-92 cells present a valuable model for the assessment of functional studies for FHL2. Studies on primary patient samples may further delineate the interactions between NK cells and MSCs, if available, in FHL2.

## Conclusion

**Fig. 6:**
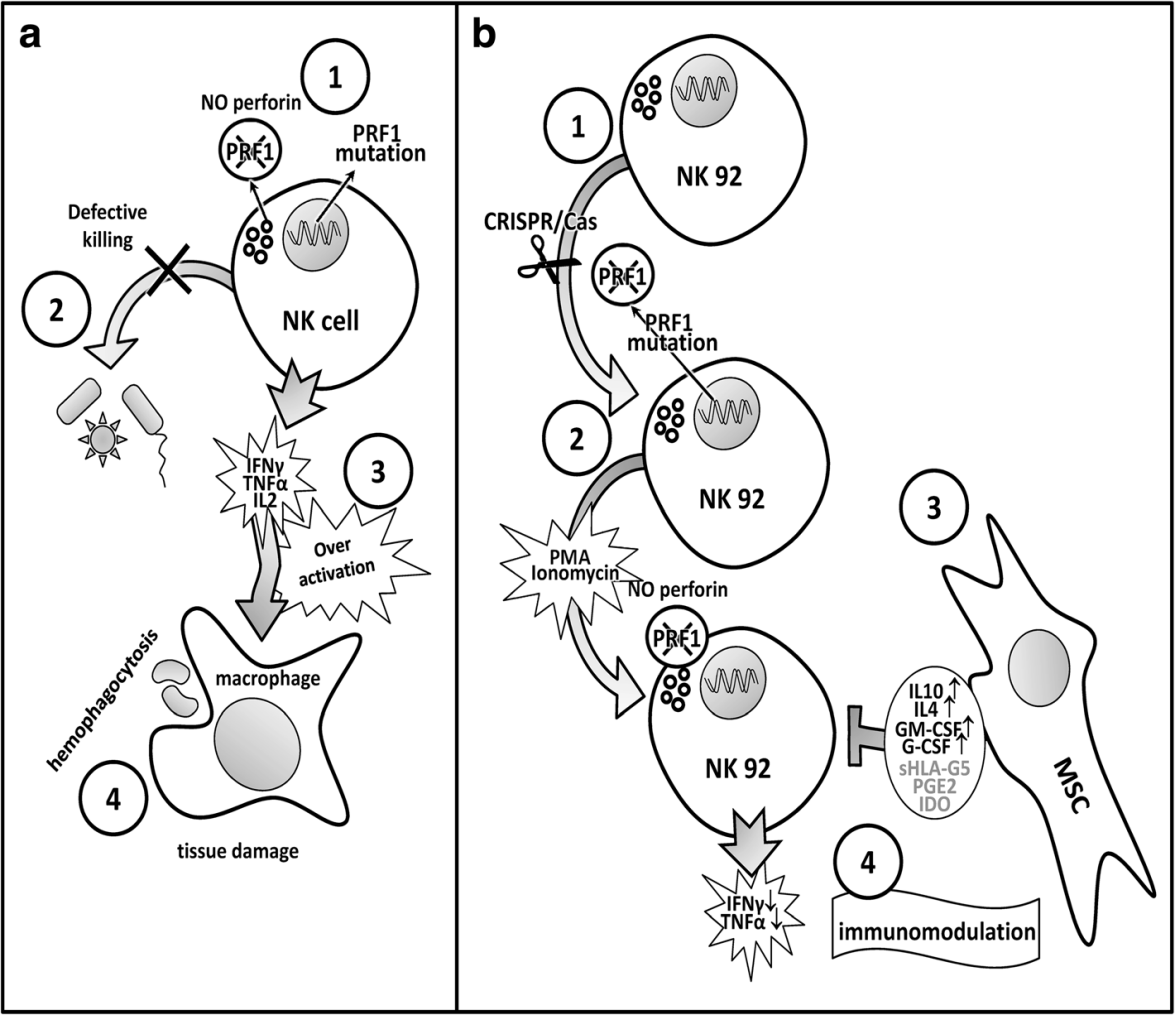
(**a**) Principle physiopathology of FLH2. Homozygous mutations in *PRF1* gene cause loss of perforin protein in lytic granules of immune cells such as NK and cytotoxic T cells (1). This functional loss leads to defective killing of infection agents (2) and causes overactivation and expansion of immune cells, concomitant with high level secretion of cytokines (3). Activated lymphocytes and macrophages infiltrate tissues, causing tissue damage as well as spontaneous phagocytosis of blood cells (4). (**b**) FLH2 in-vitro model employed in this study. NK92 cells engineered using CRISPR/Cas genome editing approach to target* PRF1* gene exon 2 (1). Selected clones defective for perforin induced with PMA and ionomysin (2), and cocultured with and without MSCs (3). Among several soluble immunomodulatory factors secreted by MSCs, IL-10 and IL-4 are induced in coculture supernatant concomitantly with diminished release of proinflammatory cytokines IFN-γ and TNF-α from NK92 cells (4). G-CSF granulocyte-stimulating factor, GM-CSF granulocyte–macrophage-stimulating factor, IDO indoleamine 2,3-dioxygenase, IL interleukin, IFN interferon, MSC mesenchymal stem cell, NK natural killer, PGE2 prostaglandin E2, PMA phorbol myristate acetate, sHLA-G5 soluble human leukocyte antigen-G5, TNF tumor necrosis factor

We have presented in-vitro proof-of-concept results for the ameliorating effect of MSCs as an adjuvant immune modulator for therapy of FHL2 patients. Schematic summaries of the FLH2 pathogenesis and the impact of MSCs shown in this work are presented in Fig. 6. This study describes an in-vitro genetically engineered human cell model that is an appropriate and valid model for future investigations of FHL2. In conclusion, this work contributes to the current knowledge base on the use of MSCs as a supportive therapy for FHL2 patients under convenient circumstances where prolonged immunosuppression is required to gain time before allogeneic hematopoietic stem cell transplantation.

## Additional files

Additional file 1: **Figure S1.** Flow cytometric immunophenotyping data of MSCs. MSCs positive for CD29 (99.5%), CD44 (99.9%), CD73 (99%), CD90 (98%), and CD105 (99%) expression, compatible with mesenchymal origin; negative for CD34 (0%), CD45 (0%), and CD3 (0%), excluding hematopoietic origin. Assay conducted on Becton Dickinson FACS Aria instrument. PE phycoerythrin, FITC fluorescein isothocyanate. **Figure S2.** Results of differentiation studies. Alizarin Red staining shows osteogenic differentiation of MSCs following 21 days in culture, 10× (**a**). Oil red O staining confirmed adipogenic differentiation and accumulation of lipid droplets after 21 days in culture, 2× (**b**). Scale bar = 100 μm. Olympus IX70, Olympus DP71 digital camera. (DOCX 1056 kb)

## Abbreviations

FHL2: Familial hemophagocytic lymphohistiocytosis 2
HLH: Hemophagocytic lymphohistiocytosis
MSC: Mesenchymal stem cell
NK: Natural killer
PMA: Phorbol myristate acetate
