# Supplementary material for: Bone marrow derived mesenchymal stem cells ameliorate inflammatory response in an in vitro model of familial hemophagocytic lymphohistiocytosis 2
Source: Stem Cell Res Ther. 2018 Jul 18;9:198. doi: 10.1186/s13287-018-0941-y (PMC6052587; doi:10.1186/s13287-018-0941-y)
Supplement: Supplementary file 1 — Figure S1. Flow cytometric immunophenotyping data of MSCs. MSCs positive for CD29 (99.5%), CD44 (99.9%), CD73 (99%), CD90 (98%), and CD105 (99%) expression, compatible with mesenchymal origin; negative for CD34 (0%), CD45 (0%), and CD3 (0%), excluding hematopoietic origin. Assay conducted on Becton Dickinson FACS Aria instrument. PE phycoerythrin, FITC fluorescein isothocyanate. Figure S2. Results of differentiation studies. Alizarin Red staining shows osteogenic differentiation of MSCs following 21 days in culture, 10× (a). Oil red O staining confirmed adipogenic differentiation and accumulation of lipid droplets after 21 days in culture, 2× (b). Scale bar = 100 μm. Olympus IX70, Olympus DP71 digital camera. (DOCX 1056 kb) [file 13287_2018_941_MOESM1_ESM.docx]

**Additional file 1:**

**Characterization of MSCs:**


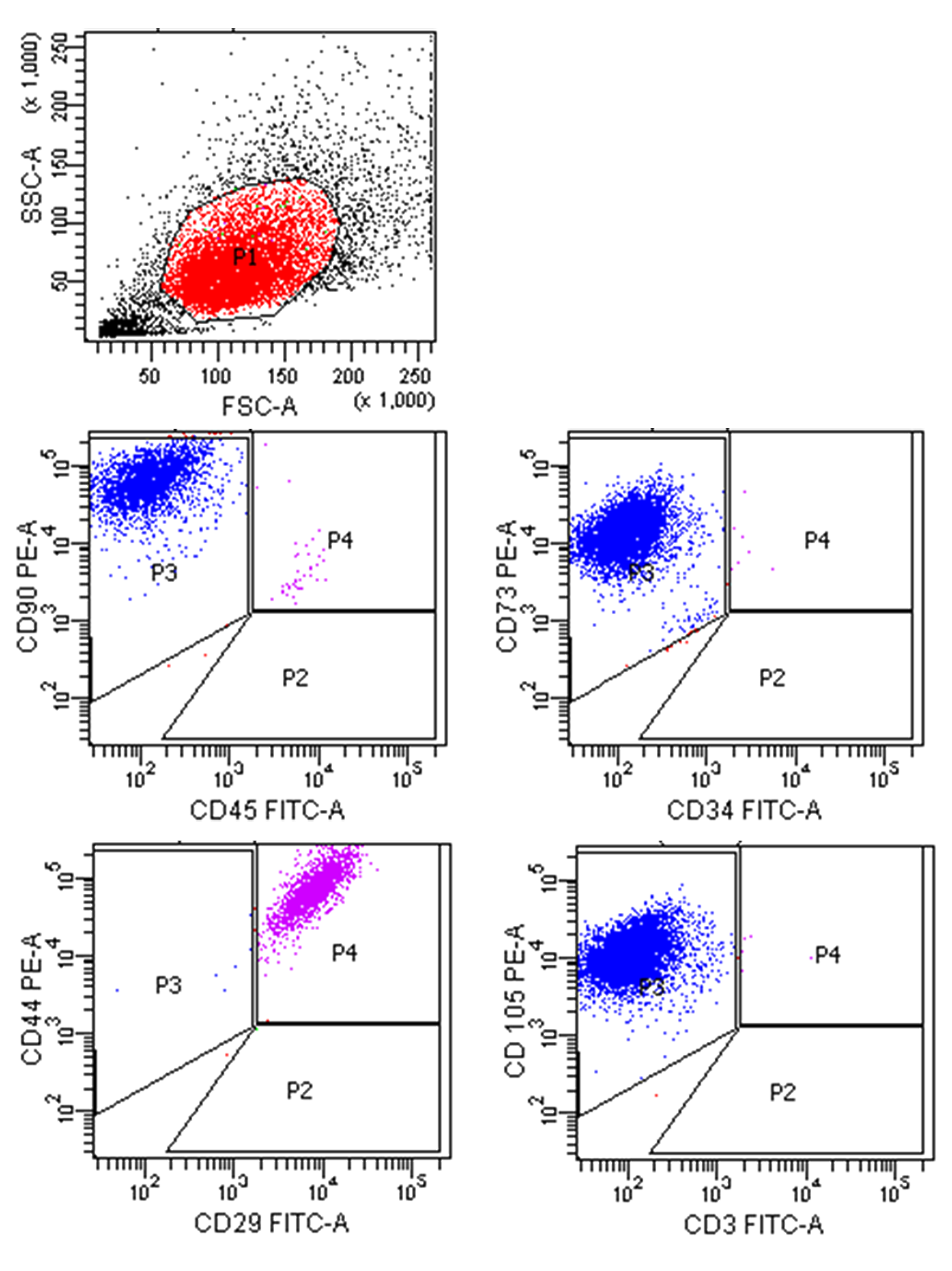


**Figure S1.** Flow cytometric immunophenotyping data of the MSCs are presented. The MSCs are positive for CD29 (99,5%), CD44 (99,9%), CD73 (99%), CD90 (98%), CD105 (99%) expression compatible with mesenchymal origin; and negativity of CD34 (0%), CD45 (0%) and CD3 (0%) excluded hematopoietic origin. PE is phycoerythrin, FITC is fluorescein isothocyanate. Assay was conducted on Becton Dickinson FACS Aria instrument.


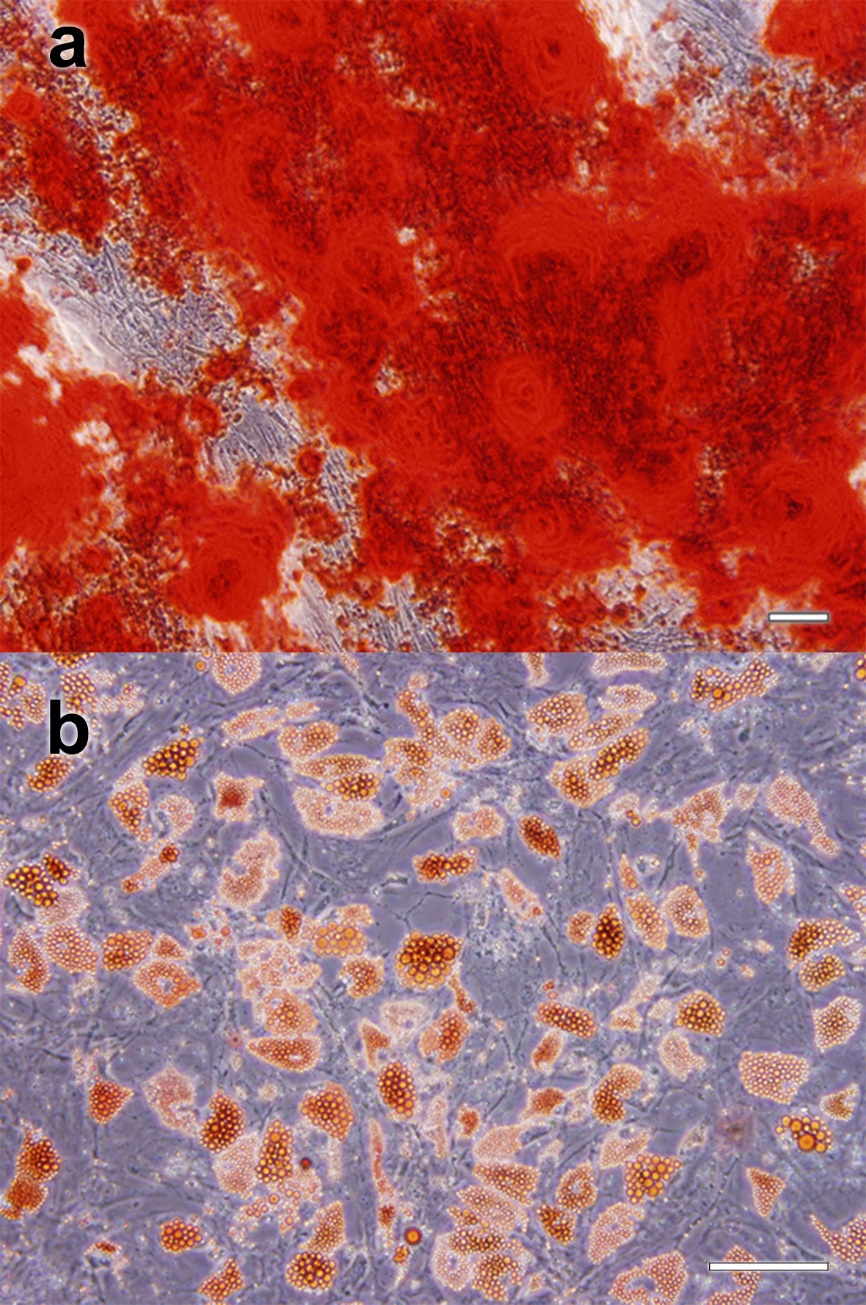


**Figure S2.** Results of the differentiation studies are presented. Alizarin red staining was done to show osteogenic differentiation of MSCs following 21 days in culture, 10X, (a). Oil red O staining confirmed the adipogenic differentiation and accumulation of lipid droplets after 21 days in culture, 20X (b). (Scale bar indicates 100 $\mu$m, Olympus IX70, Olympus DP71 digital camera)
